# Some Notes on Examinations for Life Assurance

**Published:** 1905-03

**Authors:** R. Shingleton Smith

**Affiliations:** Consulting Physician to the Bristol Royal Infirmary, and Emeritus Professor of Medicine, University College, Bristol


### SOME NOTES ON EXAMINATIONS FOR LIFE ASSURANCE.

BY

R. Shingleton Smith, M.D.Lond., F.R.C.P.,

*Consulting Physician to the Bristol Royal Infirmary, and Emeritus Professor of Medicine, University College, Bristol.*

---

It is commonly supposed that any qualified medical man is competent to undertake life insurance examinations, and many no doubt undertake them with little sense of the nature of their duty to the office by whom they are employed, often with no previous training, and with little knowledge of the problems which they may be called upon to solve. Of late, however, a tendency has been growing in favour of the idea that a life insurance examiner must be a universal specialist, and that no report can be considered complete unless the physical examination of the applicant has included the use of pretty well all the special methods of diagnosis known to the specialists in the various departments of medicine and surgery. When we read that "no means of investigation which would demonstrate the truth, by any diagnostic device whatever, should be omitted, no instrument of precision should be left unused, no matter how troublesome that use might be to the examiner, no diagnostic device should be passed untried, if by aid of instrument or art *one single fact* could be established with certainty which otherwise was only probable,"[1] &c., we cannot but wonder who will be competent for these things.

We all recognise the fact that the examination of a candidate for insurance is a problem in diagnosis, and often by no means an easy one, even after many years of experience; it is also a problem in prognosis, a still less easy one, demanding the most careful consideration; it involves also the recognition not only of disease which is, but of disease which is to be in the future,

[1] *Med. Exam. and Pract.*, 1904, xiv. 459.

and these forecasts demand the highest knowledge and the highest intellectual faculties. To make such an examination and to solve the problem which may arise must take time, and must weary the patience not only of the examiner, but of the examined; accordingly we cannot but think that an intermediate course is the only one possible, and that special examinations—microscopic, ophthalmoscopic, laryngoscopic, otologic, sphygmographic, hæmatoscopic, endoscopic, bacteriologic, and so on—must be left to the judgment of the individual examiner, who will apply them in case of need. However this may be, I read[1] "that the time is coming, nay the hour is already sounded by the clock, when those men who cannot, from lack of training, or from lack of perception of need and duty, be brought to use every means and method of diagnosis in making examinations will cease to be medical examiners." Such views as these will not commend themselves, and will not conduce to the increase of insurance, inasmuch as few candidates will be found ready to submit to such an inquisition.

Now, as regards the examination itself, the insurance offices provide forms for the guidance of the medical examiner. We sometimes wish that the form provided might be a blank sheet of paper: the experienced examiner would often give a better report in this way than by answering a long series of questions, many of which have no bearing on the case in hand. We must not, however, forget that there can be no uniformity in the results unless the various examiners have some such form for their guidance, and that the principal function of the examiner is to ascertain the facts and report the same to the medical officer-in-chief at the head office. It is true he is commonly asked to do more than this, as his opinion on the risk is also desired when he is asked to advise the actuary who knows much better; but the summing up of the whole evidence on the case and the adjudication thereon can only be done by the central authority from an examination of the collected reports of the medical examiner, the agents, the reports of confidential friends, and the opinion of the chief medical adviser.

[1] *Ibid.*

The forms commonly provide that the essential points shall not be overlooked: they are as follows:—

A.—The family history—heredity.

B.—The past medical history of the proposer.

C.—The results of the physical examination at present.

D.—The general appearance of the candidate.

E.—The occupation, habits, residence and general environment.

It is scarcely needful to observe that as a rule an ordinary case presents no difficulty. The first-class life on the one hand, and the manifestly uninsurable on the other hand, are easily determined; but the intermediate cases are those which will tax all the powers of the examiner. Here we have a great variety of problems to solve—those relating to heredity, the effects of previous illnesses, the probability of their recurrence, diminishing or increasing risks as well as present ones, and the exact value of a personal defect with its results in the future. On all those points the examiner has to give information to the actuary, who alone is qualified to determine what is the money equivalent of the defects reported. It is obvious that even with the first-class life no one is spotless: there is always some risk that the possibility of attaining the age of 98—the limit when, according to the British Life Offices, everyone ought to be dead—may be shortened in some unforeseen way. The contract for assurance payable at 98 or at previous death is accepted by the offices on their own conditions.

These conditions are grounded on the duration of life of the normal man, of good parentage and with a healthy environment. This is the requisite standard, but how few of us attain to it. The risk accepted by the office is a great one, for who can predict the accidents of contagion, who can forecast a fatal attack of pneumonia? Many years ago I was asked to examine a spare man with a family history of phthisis. In spite of my recommendation to accept the risk it was declined. Fifteen years later the proposal was renewed, the applicant being quite well and having had no illness during the interval. The risk was then accepted, the first premium paid, death occurred from pneumonia a few months later, and the widow derived much benefit from the two thousand pounds on which one premium only had been paid. It should be generally admitted that every person should be allowed the privilege of insurance unless good cause to the contrary can be shown. The object of the medical examiner is not to endeavour to reject an applicant, but whilst safeguarding the company for which he acts, he should not decline the risk without very good reason, and should not expect that every candidate for insurance should attain the standard of the first-class life both by heredity and environment. The vital stamina may be deficient or the environment adverse, and yet that particular person may attain a good age and prove to be a profitable client. I was once told by a robust-looking gentleman of over 70 years of age that he was the only surviving member of a family of seven who had died from phthisis, and that he was in early life declined insurance because of his family history.

The most important questions of heredity relate to cancer and tuberculosis. On these two points much information is given in the most recent data of the Scottish Widows' Fund,[1] from which the following conclusions seem to be justified:—

1. The registered increase in the number of deaths from cancer is undoubted.

2. After allowing that this increase is not wholly real, but may be accounted for, to some extent, on the assumption that the true nature of obscure cases of malignant disease has been recognised with ever-increasing certainty in recent years, and that, as a consequence, the statement of death has been made with greater precision than had been formerly the case, there remains a large, real increase to account for the large and progressive mortality from this disease.

3. The age period at which death from cancer is most frequent is gradually declining according to the Scottish Widows' Fund returns.

4. The average age at death from cancer among our members declined by two years from 1874–80 to 1888–94, as contrasted with a rise in the average age at death from all causes of a little over two years.

1 "The Causes of Death among the Assured in the Scottish Widows Fund, 1874–1894," reported by Claud Muirhead, M.D.

5. The office returns mark a *decrease* in deaths from *internal* cancer of 7.70 per cent., and an *increase* in deaths from *external* cancer of 18.89 per cent. of the percentages in the first septennium as contrasted with the third.

Dr. Muirhead further remarks that "during the twenty-one years under observation, cancer as a cause of death among our members aged from 45 to 65 has made rapid and startling progress."

As regards hereditary predisposition to phthisis, it has been sought of late years to deny that such a factor exists at all, and that all cases of phthisis should be attributed to direct infection. From an examination of the data it is stated that "the logical conclusion is that, in the adjudication of a proposal with a family history of phthisis, while we cannot discard from our minds altogether such history as a predisposing cause of the disease, we must attach a much less degree of importance to it than has been the practice in former years," prior to the discovery of the tubercle bacillus. Not more than 35 per cent. of the consumptives exhibited any family predisposition to phthisis.

A history of pleurisy, as suggestive of tuberculosis, has recently assumed a grave importance, and the latest dictum is that of Dr. William Osler[1] at Oxford, who states that "during the last twenty-five years the evidence has been accumulating in favour of the tuberculous nature of a large proportion of all cases of simple sero-fibrinous pleurisy. The exact percentage is hard to determine, but, in the absence of pneumonia or sepsis, the very existence of a pleural exudate is in itself sufficient to raise a suspicion of tuberculosis."

Hæmoptysis is a non-infrequent occurrence in those who have no other evidence of lung disease, but it is rightly regarded with much apprehension, and it is usually safe to act upon the assumption that there must be more or less active disease in the lung as its cause. Of course the spitting of blood may not be hæmoptysis of the graver kind, but it is usual to decline any proposer until there has been no return of the hemorrhage for seven or ten years, after which period the

[1] *Brit. M. J.*, 1904, ii. 999.

applicant, having reached the age of 35 or 40 and being in good health, he may be accepted as a good life.

Other questions of heredity relate to insanity, diabetes, gout, cardiac conditions, and so on. Of these I remember a notable case of a young lady who, after having been a diabetic for some years, was accepted by an insurance examiner as a first-class life: within a very few weeks she died after a few hours of coma. This lady's father had died of tubercular phthisis after some ten years of diabetes, and a brother has since died from acetinæmic coma as a sequel of diabetes.

Concerning the heart, it is only to be expected that such hereditary maladies as rheumatism and gout should be frequent antecedents of heart disease. It is commonly understood that rheumatic fever in a parent predisposes not only to the same disease in the offspring, but to chronic defects of the heart, even although there may be no history of an attack of acute rheumatism. Hence the insurance offices very rightly attach great importance to the question relating to rheumatic fever.

Diseases of the nervous system must be regarded as likely to be of influence in the offspring. With a family history of well-marked neuroses it would not be safe to accept the risk unless the proposer had reached middle life and had not developed previously any indications of a neurotic tendency. The subject of insanity and epilepsy in relation to life assurance has recently been very exhaustively summarised by Sir W. R. Gowers.[1]

As regards the physical examination of the applicant, the most important and obvious questions relate to the height and weight. The men of moderate stature, ranging from 5 ft. 6 in. to 5 ft. 9 in., are considered to be the most capable, with an average weight of 10 st. 4 lb. to 11 st. 10 lb. As regards the practical estimation of these, it is interesting to note that the heels of the boots are considered to approximately counterbalance the weight of the clothing, so that the person may be measured and weighed in the usual garments in which he appears. It is commonly the rule to allow a margin of 15 or 20 per cent. on either side of the normal average as consistent

[1] *Lancet*, 1904, ii. 1061.

with good health and good constitution, but anything over this margin suggests the perils of obesity, and a marked under-weight suggests the possibility of phthisis. Any difficulty arising from over-weight, all other points being good, may be arranged by the recommendation of an endowment policy payable at a certain age, the insurance being then for a short term of fifteen or ten years. This gets rid of the fact that those of over-weight tend to die prematurely of apoplexy, heart disease, atheromatous degeneration of the blood vessels, and having poor resisting power, do not throw off the effects of serious ailments as well as those of normal weight.

Dr. Muirhead [1] remarks that "light-weights are looked upon as undesirable risks, because, being usually of nervous constitution, they take affairs of life more seriously than others, and are predisposed to early death from nervous breakdown and tubercular complaints . . ." The investigation of 524 schedules of males who had died of phthisis, when compared with 502 schedules of those who had died of apoplexy, "corroborates the popular opinion that men of light weight exhibit a special proclivity to phthisis, and that over-weight, while not excluding the possibility of the individual falling a victim to lung disease, is much more commonly associated with disease of the nervous system than with phthisis; so that very light weight in a candidate for assurance must always be regarded by the medical examiner as a danger-signal, warning him to be more than usually vigilant in his investigation of the condition of the respiratory organs."

The same author also directs attention to the fact that the prevalence of consumption is by no means confined to persons under middle life, inasmuch as phthisis is a potent cause of death amongst the older members. The statistics of the Scottish Widows' Fund directly contradict the popular belief that this disease belongs to the period of youth and young manhood, and is rarely encountered after 45 years of age.

I well remember a case illustrating this point some years ago. The wife died from phthisis at early middle age, one after another of her children followed, then the wife's sister living

[1] *Op. cit.*, p. 44.

in the same house, and last of all the father developed an attack of tubercular pleurisy at about the age of 70 from which he died after a comparatively short illness.

It has been remarked[1] that "there is more certainty about the result of examination of middle-aged and elderly applicants whose habits are formed, and who have passed through the dangers and risks of early adult life. There are, however, some special difficulties and pitfalls to be avoided in the examination of these lives."

The question is commonly asked whether the apparent age is in agreement with the age stated. Now this appears to be one of the superfluous questions which might well be deleted from the form. The answers to it must be absolutely unreliable and vitiated by the common experience that some look old whilst young and never look older, some appear to be born looking old, and others grow old but still look young. The question as to resemblance to which parent is another which cannot be of any reliable assistance, and must commonly depend upon the statement of the proposer, unverified by the medical examiner.

The chest measurement is usually asked for, but there must be many fallacious measurements in the case of women applicants encumbered with much clothing and often considerable collections of loose fat. Tables of these measurements[2] show that the medium circumference of the chest varies from 33½ inches in a man of 5 feet up to 40½ inches in a man of 6 feet. Approximately the chest measurement should not be less than half the height; that is to say, a man of 5 ft. 6 in. should measure not less than 33 inches. "A good chest expansion is a great safeguard against pulmonary tuberculosis, and a range of expansion from forced expiration to forced inspiration of 2 inches at 5 feet height and 3 inches at 6 feet height respectively should be the minimum." Women commonly weigh less than men up to a height of 5 ft. 7 in., but over this they may be more.

The problems associated with the examination of the heart

[1] Havilland Hall, *Life Assurance*, 3rd edition, 1903, p. 43.
[2] Havilland Hall, *Op. cit.*, p. 46.

are many and of the greatest importance. On the one hand there can be no doubt that many applicants are rejected on account of supposed heart disease, and *vice versâ* some heart defects may escape observation. Ephemeral murmurs may be mistaken for those of organic disease, and organic murmurs are not always indicative of serious defect. The absence of a bruit is not sufficient evidence that there is no organic defect, and the presence of a bruit does not necessarily render the proposer ineligible. Commonly the mere existence of a cardiac murmur has been considered sufficient to exclude an applicant from insurance, but of late years it has been argued that even well-marked examples of valvular disease may be accepted at an increased premium. The large variety of murmurs of varying intensity and diffusion commonly classed as hæmic would obviously suggest the postponement of the application for three or six months pending the results of treatment. A cardio-respiratory murmur at or outside the apex may be easily mistaken for that of organic disease, and has frequently been the reason for rejection of a candidate. Neither of these murmurs should be a sufficient reason for a definite refusal of the proposal. But on the other hand, if there exists a definite systolic murmur at the apex believed to be of organic origin, and even if there should be no other evidence of heart defect or of weakness of circulation, the uncertainty of prognosis in such cases must render the applicant ineligible for insurance. It must be impossible for the medical examiner to advise the office to accept the risk when a sudden heart failure is at any time a by no means remote possibility. The presence of a murmur of mitral or aortic stenosis or one of aortic regurgitation will be still more emphatic in causing rejection. In spite of the fact that cases of aortic regurgitation and of mitral regurgitation with good compensation have been known to continue for very many years, and the patients sometimes survive to a good old age, yet the uncertainty of such conditions, and the facility with which failure of the compensatory conditions may arise, must give no hesitation in absolutely declining to entertain an acceptance of such risks. A diagnosis of hypertrophy, indicated by a supposed displacement of the apex beat, has often given rise to a difficulty. Here there are many fallacies due to obliquity of ribs, and other causes which may lead the examiner into error. There are many conditions where, in the absence of a murmur, the position of the apex beat, its character and that of the sounds, the rhythm and regularity or otherwise of the heart beat, may be such that the integrity of the heart muscle may be gravely questioned: in all such a rejection of the proposal is inevitable. The cases in which fatty heart may be suspected give much ground for question, for if all cases are rejected in which the impulse is feeble and irregular, it will lead to the loss to the company of many good lives.

Of the pulse-rate there can be no rigid limit. Nervousness is not confined to women, and a quick pulse is often the result of many trivial and temporary causes. Cases of persistent tachycardia or bradycardia should lead to inquiry as regards the possibility of brain lesions, of arterio-sclerosis, fatty heart, Graves' disease, or excess of alcohol and smoke.

In connection with diseases of the arteries and aneurism it is well to remember that arterial murmurs of an ephemeral character may be due to the pressure of unusually well-developed muscles. It was once my duty to report on an eminent cricketer who had been declined insurance in consequence of a murmur below the clavicle, which was no doubt due to this cause. There was no other defect, and the risk was duly accepted.

The state of the genito-urinary organs presents many questions. Syphilis and gonorrhœa appear to be remarkably rare in applicants for insurance, and hence the need for critical clinical observation on the part of the observer, and especially as to the veracity of the applicant. Of course no case can ever be accepted so long as sugar or albumen is found in the urine, but a temporary glycosuria or a temporary albuminuria would not permanently exclude the candidate from insurance. A passing glycosuria in middle life is quite as common as what is called cyclic albuminuria in the youth, and although it cannot be discreet to accept the risk in the presence of either, the disability may be only temporary and need postponement of the proposal for three or six months.

The general appearance of the proposer presents many points for observation: anæmia, or cyanosis, injected capillaries of the cheek, œdema of eyelids, an icteric tint, or contracted pupils, the condition of the tongue, throat, gums, and teeth, the proportion of muscle to fat, and the patient's bearing under examination, as unusual reticence and sometimes a craving for morbid accuracy may give information of an important character. All these points should be passed in mental review and noted when needful.

The older forms commonly make an inquiry as to the temperament of the proposer. Now the modern medical man is not educated in the classical temperaments, and has little regard for them. If he attempts an answer to the question he does so in a casual kind of way, deeming it of no importance. Dr. Stillman [1] has the audacity to devise a new series of temperaments of his own. He cuts down the classical four—lymphatic, sanguine, bilious and nervous—to three, and those he designates on an anatomical basis—motive, vital and mental. He remarks that "when either of the temperaments exists in excess, the result is necessarily a departure from symmetry and harmony both of mind and body, the one always affecting the character and action of the other. Perfection of constitution consists in a proper balance of temperaments, and whatever tends to destroy this balance should be avoided." We are told that in this country diverse nationality and parentage render it more and more difficult to find typical specimens of the normal temperaments, and that the same individual at different ages may be classified under different headings. According to age, the child approximates the sanguine or vital temperament; the mature adult the bilious; while advancing age produces a tendency either to excess of the nervous or phlegmatic elements. We think that the whole question of temperaments, and the supposed liability to particular forms of disease associated therewith is so beset with fallacies that they are of little use in estimating the probable duration of life or liability to disease.

The words diathesis and cachexia are also of little use from

[1] Stillman, *Life Insurance Examiner*, 1888.

the insurance standpoint. Few cases with such chronic ill-health as would justify the appellation "cachectic" are likely to apply for insurance, and these would be at once recognised as ineligible. As regards the strumous, gouty and rheumatic diatheses, there will be information derived from the family history.

The information as regards the vital statistics of the family is at all times a little difficult to obtain; the want of knowledge and the fallacies of memory greatly test the patience and the tact of the examiner. It is not surprising that people know so little of the causes of death of their grandparents, whom often they have never seen and of whom they have rarely heard; beyond the father and mother the information can only be approximate, but it becomes of importance when either parent has died in early life. It is also not to be wondered at that many applicants know very little of the cause of death of elder brothers and sisters whom they may have never seen, and of whose diseases they can personally know nothing. Any person of intelligence should be able to state the cause of death of brothers and sisters who have been known to them, but it is not surprising that the non-medical mind should often fail in giving anything like an accurate statement of events which may have occurred many years previously. The examiner has to be cautious in the acceptance of these statements, and especially when there is method in the want of knowledge and apparent want of memory which may be more feigned than real.

We often hear of insurance without medical examination, and of the alleged uselessness of medical selection. No doubt there are many fallacies into which the most careful examiner may fall, and the most honest judgment may be neutralised by the unavoidable accidents and uncertainties of life, but it has been shown by the statistics of the Scottish Widows' Fund[1] that an enormous disproportion is observed between the rates of mortality from consumption of those insured and those recorded by the Registrar-General. The deaths from consumption at all ages amongst the selected lives were 81.15 per 10,000 as compared with 178.29 during the ten years 1881–90, as given

[1] *Op. cit.*, p. 39.

by the Registrar-General. This disproportion is clearly due to the medical examination which each candidate has to undergo before being admitted to the benefits of insurance. It is also scarcely necessary to remark that the mere presence of a medical examination prevents the application of the large body of persons who are known to have organic and other defects which would render their acceptance improbable. No one likes to be rejected, and few will make any overtures if they have any suspicion that they may not be acceptable.

Much disputation has existed from time to time as regards the fee which the offices should pay for the medical service rendered. It has always appeared to me that there can be no rational excuse for the practice of cutting down the fee to one-half because the amount to be insured is small. The examination is the same whatever the amount of the insurance may be. No honest medical examiner can proportion his examination to the size of the fee or to the sum insured. This will always take a considerable time and require the same careful consideration, and it is not necessary that the medical examiner should know whether the proposal is for one hundred or ten thousand pounds. So also as there is no reason for cutting down the fee for the smaller sums, neither is there any for doubling it in the case of the larger amount. It is true the responsibility appears to be greater; but this should have no influence in the examination, which is presumed to be in all cases complete, thorough and honest.

